# Supplementary material for: Macromolecular Brushes Based on Poly(L-Lactide) and Poly(ε-Caprolactone) Single and Double Macromonomers via ROMP. Synthesis, Characterization and Thermal Properties
Source: Polymers (Basel). 2019 Oct 1;11(10):1606. doi: 10.3390/polym11101606 (PMC6835319; doi:10.3390/polym11101606)
Supplement: Supplementary file 1 [file polymers-11-01606-s001.docx]

**Macromolecular Brushes Based on Poly(L-Lactide) and Poly(ε-Caprolactone) Single and Double Macromonomers via ROMP.**

**Synthesis, Characterization and Thermal Properties**

**Christiana Nikovia, Eleftheria Sougioltzoupoulou, Vyron Rigas, Marinos Pitsikalis***

Industrial Chemistry Laboratory, Department of Chemistry, National and Kapodistrian University of Athens, Panepistimiopolis Zografou, 15771 Athens Greece

***** Correspondence: [pitsikalis@chem.uoa.gr](mailto:pitsikalis@chem.uoa.gr); Tel.: +30-210-727-4440

**Supporting Information Section**

**Figure S1.** ^1^H NMR spectrum of sample SDBC in CDCl_3_.

**Figure S2.** ^1^H NMR spectrum of sample BDBC in CDCl_3_.

**Figure S3.** ^1^H NMR spectrum of sample B-(PLLA-b-PCL-b-PLLA) in CDCl_3_.

|  |  |
| --- | --- |

**Figure S4**. DTG plot of the samples NBE-PLLA and NBE-PCL

**Figure S5**. DTG plot of the samples NBE-(PLLA)_2_

**Figure S6**. DTG plot of the samples (NBE-PCL)_2_

**Figure S7**. DTG plot of the sample DB-PCL

**Figure S8**. DTG plot of the sample SDBC

**Figure S9.** DTG plot of the sample DB-PLLA

**Figure S10**. DSC graph for the double macromonomer NBE-(PCL)_2_

Figure S11. DSC graph for the double brush DB-PCL

**Figure S12**. DSC graph for the double macromonomer NBE-(PLLA)_2_
